# Expanding the clinical phenotype associated with *NIPAL4* mutation: Study of a Tunisian consanguineous family with erythrokeratodermia variabilis—Like Autosomal Recessive Congenital Ichthyosis

**DOI:** 10.1371/journal.pone.0258777

**Published:** 2021-10-20

**Authors:** Cherine Charfeddine, Nadia Laroussi, Rahma Mkaouar, Raja Jouini, Olfa Khayat, Aladin Redissi, Amor Mosbah, Hamza Dallali, Achraf Chedly Debbiche, Anissa Zaouak, Sami Fenniche, Sonia Abdelhak, Houda Hammami-Ghorbel

**Affiliations:** 1 University Tunis El Manar, Institut Pasteur de Tunis, Biomedical Genomics and Oncogenetics Laboratory, LR20IPT05, Tunis, Tunisia; 2 Université de la Manouba, Institut de Biotechnologie de Sidi Thabet, Ariana, Tunisia; 3 Department of Pathology, Habib Thameur Hospital, Tunis, Tunisia; 4 BVBGR-LR11ES31, ISBST, Université de la Manouba, Institut de Biotechnologie de Sidi Thabet, Ariana, Tunisia; 5 Department of Dermatology, Genodermatosis and Cancers Laboratory LR12SP03, Habib Thameur Hospital, Tunis, Tunisia; University of Iowa, UNITED STATES

## Abstract

Erythrokeratodermia variabilis (EKV) is a rare disorder of cornification usually associated with dominant mutations in the *GJB3* and *GJB4* genes encoding connexins (Cx)31 and 30.3. Genetic heterogeneity of EKV has already been suggested. We investigated at the clinical and genetic level a consanguineous Tunisian family with 2 sisters presenting an autosomal recessive form of EKV to better characterize this disease. Mutational analysis initially screened the connexin genes and Whole-exome sequencing (WES) was performed to identify the molecular aetiology of the particular EKV phenotype in the proband. Migratory shaped erythematous areas are the initial presenting sign followed by relatively stable hyperkeratotic plaques are the two predominates characteristics in both patients. However, remarkable variability of morphological and dominating features of the disease were observed between patients. In particular, the younger sister (proband) exhibited ichthyosiform-like appearance suggesting Autosomal Recessive Congenital Ichthyosis (ARCI) condition. No causative mutations were detected in the *GJB3* and *GJB4* genes. WES results revealed a novel missense homozygous mutation in *NIPAL4* gene (c.835C>G, p.Pro279Ala) in both patients. This variant is predicted to be likely pathogenic. In addition, *in silico* analysis of the mutated 3D domain structure predicted that this variant would result in NIPA4 protein destabilization and Mg^2+^ transport perturbation, pointing out the potential role of *NIPAL4* gene in the development and maintenance of the barrier function of the epidermis. Taken togheter, these results expand the clinical phenotype associated with *NIPAL4* mutation and reinforce our hypothesis of *NIPAL4* as the main candidate gene for the EKV-like ARCI phenotype.

## Introduction

The Erythrokeratodermias are refers to a heterogeneous group of rare inherited disorders of cornification characterized by erythematous, hyperkeratotic, sharply demarcated plaques, which are often transient or migratory [1]. Clinically, erythrokeratodermias can be divided into two main groups, erythrokeratodermia variabilis (EKV) and progressive symmetric erythrokeratodermia (PSEK), despite phenotypic overlaps reported between these two entities [2]. However, there is a debate as to whether these two presentations are distinct entities and, later, the designation of erythrokeratodermia variabilis et progressiva (EKVP, MIM 133200) has been proposed to encompass the diversity of the clinical phenotypes of both EKV and PSEK (van Steensel 2004). The hallmark of the EKV (is the occurrence of transient erythematous patches and localized or generalized hyperkeratotic plaques, both of which might appear and regress within minutes to hours [3, 4]. Palmoplantar keratosis is often present, characterized by fine erythematous scaling rather than the thick hyperkeratosis. The condition tends to present at birth or during the first year, more rarely occurs later in childhood and even in early adulthood [5]. Because of its rarity and considerable clinical variability, the classification of EKV tends to be difficult particularly as there is phenotypic overlap with other erythrokeratodermas and also the existence of several rare variants of EKV [6–9]. The disease is usually inherited in an autosomal dominant trait. Mutations in Connexin (Cx) genes, *GJB3* (Cx31) and *GJB4* (Cx30.3) have been identified in most families or sporadic cases presenting classical EKV phenotype [10–16]. So far, only two recessive cases, both caused by homozygous mutations in *GJB3*, have been reported in patients with EKV [15, 16]. However, the EKV disorder is clinically heretogenous even among patients harboring the same *GJB3* or *GJB4* mutations. In addition, same disease-causing *GJB4* or *GBJ3* mutations may cause either an EKV or a PSEK, make challenging the clinical and molecular diagnosis of these conditions [17–19]. More recently, the genotypic landscape of EKV has been extended by the application of next-generation sequencing (NGS) and it has been demonstrated that mutations in ichthyosis-related genes involving *ABHD5*, *ELOVL4* and *PNPLA1* are associated with rare clinical variants of EKV or EKV-like syndromes that can occur with or without typical clinical presentation of ichthyosis [20–22]. This observation demonstrates the extensive genetic heterogeneity of EKV. Nevertheless, the molecular aetiology of many other EKV cases do not have identifiable pathogenic mutations in the two mainly involved epidermal connexin genes and likely represent a heterogeneous group of other disorders that remain to be better defined on a clinical and molecular level [7, 23, 24]. In this paper, we describe the clinical phenotype and molecular analysis of a consanguineous Tunisian family with two patients presenting EKV phenotype in autosomal recessive inheritance pattern without mutations in the *GJB3* and *GJB4* genes. The clinical characterization of the younger patient (the proband) highlights the presence of ichthyosiform-like lesions phenotypically. Exome sequencing reveals a novel homozygous likely pathogenic variant in *NIPAL4* gene, that might underlines the EKV-like Autosomal Recessive Congenital Ichthyosis (ARCI) phenotype in this family. So far, pathogenic variants in *NIPAL4* have been associated with ARCI phenotypes. Our study shows the utility of NGS in unravelling the molecular aetiology of rare diseases with genetic heterogeneity and overlapping phenotypes.

## Patients and methods

### Patients and material

This study was conducted according to the principles of the declaration of Helsinki and approved by the biomedical ethics committee of Institut Pasteur de Tunis (2017/31/I/LR16IPT05).

A consanguineous family designed (EKV-ICH1) including two affected siblings, their unaffected mother and brother were enrolled in this study. Verbal and written informed consent was obtained from the mother.

Genomic DNA was extracted from peripheral blood leucocytes from the proband case, the affected sister and 2 unaffected family members using standard extraction procedures [25].

A 3-mm punch biopsy specimen of one of the plaques on the upper right thigh was taken for histological examination from the proband.

### Genetic investigation

#### Screening *GJB3* and *GJB4* genes

The proband and affected sister from family (EKV-ICH1) were assessed for disease-causing mutations in *GJB3* and *GJB4* genes. PCR conditions and direct sequencing analysis of the exon and flanking introns regions of *GJB3* (NM_024009) and *GJB4* genes (NM_153212) were conducted as described [12, 26].

#### Whole-exome sequencing and bioinformatic analysis

Whole exome sequencing (WES) was performed for the proband (EKV-ICH1.2) by IntegraGen (Evry, France). Exome was captured from genomic DNA using Agilent SureSelect Human All Exon V5 kit d. Libraries were sequenced on an Illumina HiSeq 2000 sequencer using 100-bp paired-end reads [26].

Alignment to the human genome reference sequence was carried out using the Burrows-Wheeler Aligner-MEM (BWA-MEM version 1.1.1; https://bio-bwa.sourceforge.net). The human genome reference version GRCh38 was used to align Fastq files. Duplicates were removed from BAM files using PICARD tool (www.picard.sorceforge.net). Genome Analysis Tool Kit (GATK, www.broadinstitute.org/gatk/) was used for Indels realignment by the Realigner Target Creator tool. GATK was also used for base quality score recalibration. Variant Calling was performed by the GATK package Haplotype Caller.

Variants were filtered to include only those with >20× read depth and mapping quality score > = 30. Functional annotation of variants wascarried out using VarAft software version 2.06 (varaft.eu/index.php).

The likely pathogenicity of annotated genetic variants was evaluated according to the following pipeline: 1-Synonymous genetic variants were filtered out, keeping only nonsense, missense, frameshift insertions/deletions and splice-site variants, as they are more likely to have functional effect. 2-Genetic variants with minor allele frequency (MAF) >1% in the Exome Aggregation Consortium (ExAC.BroadInstitute.org), the 1,000 Genomes Database (thousandgenomes.org), and gnomAD (gnomad.broadinstitute.org) were excluded. 3-Deleteriousness prediction of the missense variants was assigned using several bioinformatics programs in VarAft including MutationTaster (mutationtaster.org/; PolyPhen (genetics.bwh.harvard. edu/pph2/), fathmm (http://fathmm.biocompute.org.uk), SIFT (sift.bii.a-star.edu.sg/), PROVEAN v1.1 (provean.jcvi.org/), and MutationAssessor 1.0 (mutationassessor.org/r3/), MetaLR, MetaSVM and Combined Annotation Dependent Depletion (CADD) (http://cadd.gs.washington.edu/home). The potential effect of splice-site variants was predicted *in silico* by the Human Splicing Finder [27].

Variants were interpreted and classified according to the guidelines from the American College of Medical Genetics and Genomics (ACMG) [28]. Variants classified as pathogenic and likely pathogenic were also considered causative when in accordance with the inheritance pattern and clinical presentation.

Among all potential pathogenic variants identified in the current datasets, we mainly focused on variants in known causative genes involved in Autosomal Recessive Congenital Ichthyosis (ARCI) based on the clinical overlapping features with the proband’s phenotype, those related to EKVP based on the phenotypic heterogeneity of the erythrokeratodermias, as well as, genes associated with the differential diagnosis of EKVP based on the previously cases reports [12, 29–32].

According to the Online Mendelian Inheritance in Men database (https://www.ncbi.nlm.nih.gov/omim/) and the literature review, we established a list of the most common mutated genes in ARCI, EKVP and the differential diagnosis of EKVP that might contribute to the disease phenotype of the proband in family EKV-ICH1. The list of prioritized candidate genes included 14 genes and one disease locus interval on 12p11.2–q13 known to be associated with ARCI phenotypes, 8 EKVP related-genes and 6 genes were considered in the differential diagnosis of EKVP. The full list of genes and their associated diseases screened in this work can be found in Table 1. If no significant sequence variations were detected in the selected genes, we extended the mutational search to all WES data.

**Table 1:** List of potential disease causative genes of EKV phenotype of the proband (EKV-ICH1.2).

Disease phenotype	Phenotype (MIM)	Inheritance mode	Gene	Locus	Protein/enzyme
**Autosomal Recessive Congenital Ichthyosis associated genes**					
ARCIA4A	601277	AR	*ABCA12*	(2q34)	ATP-binding cassette sub-family A member 12 (ABCA12)
ARCI4B (HI)	242500				
ARCI2	242100	AR	*ALOX12B*	(17p13.1)	12R-lipoxygenase (12R-LOX)
(LI), (CIE)					
ARCI3	606545	AR	*ALOXE3*	(17p13.1)	Epidermal lipoxygenase-3 (eLOX-3)
(LI), (CIE)					
ARCI12	617320	AR	*CASP14*	(19p13)	cysteinyl aspartate-specific proteinase 14 (CASP14)
ARCI9	615023	AR	*CERS3*	(15q26.3)	Ceramide synthase-3 (CERS-3)
ARCI5	604777	AR	*CYP4F22*	(19p13.12)	Cytochrome P450 4F22 (CYP4F22)
(LI)					
ARCI8	613943	AR	*LIPN*	(10q23.31)	Lipase member (LIPN)
(LI)					
ARCI6	612281	AR	*NIPAL4*	(5q33.3)	Magnesium transporter NIPA4 (NIPAL4, ichthyin)
(LI), (CIE), (CI)					
ARCI10	615024	AR	*PNPLA1*	(6p21.31)	Patatin-like phospholipase domain-containing protein 1 (PNPLA1)
(CI), (LI)					
ARCI13	617574	AR	*SDR9C7*	(12q13.3)	Short-chain dehydrogenase/reductase family 9C member 7 (SDR-O)
Ichthyosis prematurity syndrome	608649	AR	*SLC27A4*	(9q34.11)	Long-chain fatty acid transport protein 4 (FATP4)
ARCI14	617571	AR	*SULT2B1*	(19q13.33)	Sulfotransferase family 2B member 1 (SULT2B1)
ARCI11	602400	AR	*ST14*	(11q24.3)	Transmembrane Serine Protease Matriptase (ST14)
ARCI1	242300	AR	*TGM1*	(14q11.2)	Transglutaminase-1 (TGm-1)
(LI), (CIE)					
ARCI7	615022	AR	-	(12p11.2-q13.1)	-
**Erythrokeratoderma variabilis et progressive (EKVP)**					
ISQMR	614457	AR	*ELOV4*	(6q14.1)	Elongation of very long chain fatty acids 4
EKVP3	617525	AD	*GJA1*	*(6q22*.*31) *	Connexin 43 (Cx43)
EKVP1	133200	AD, AR	*GJB3*	*(1p34*.*3)*	Connexin 31 (Cx30.3)
EKVP2	617524	AD	*GJB4*	*(1p34*.*3)*	Connexin 30.3 (Cx30.3)
EKVP4	617526	AR	*KDSR*	(18q21.33)	3-ketodihydrosphingosine reductase
EKVP5	617756	AD	*KRT83*	(12q13.13)	Keratin 83
EKVP7	619209	AR	*PERP*	(6q23.3)	p53/p63 tetraspan
EKVP6	618531	AD	*TRPM4*	(19q13.33)	Transient receptor potential cation channel, subfamily M, member 4
**Differential diagnosis of EKVP**					
Chanarin-Dorfman syndrome	275630	AR	*ABHD5*	(3p21.33)	Abhydrolase domain containing 5 (ABDH5)
KID	148210	AD	*GJB2*	(13q12.11)	Connexin 26 (Cx26)
Annular epidermolytic ichthyosis	607602	AD	*KRT1/KRT10*	(12q13.13-7q21.2)	Cytokeratin 1/Cytokeratin 10
Loricrin keratoderma	604117	AD	*LOR*	(1q21.3)	Loricrin
Netherton syndrome	256500	AR	*SPINK5*	(5q32)	Serine protease inhibitor Kazal-type 5 protein (LEKTI)

AR: autosomal recessive, AD: autosomal dominant

Genes are listed in alphabetic order.

LI: lamellar ichthyosis, CIE: congenital ichthyosiform erythroderma, ARIH: autosomal recessive ichthyosis with hypotrichosis, IFAH: ichthyosis and follicular atrophoderma with hypotrichosis, EKVP: erythrokeratodermia variabilis et progressiva, ISQMR: Ichthyosis, spastic quadriplegia, and mental retardation.

Reference sequences (GenBank): ABCA12 (NM_173076); ALOX12B (NM_001139); ALOXE3 (NM_001165960); CASP14 (NM_012114); CERS3 (NM_001378789); CYP4F22 (NM_173483); LIPN (NM_001102469); NIPAL4 (NM_001099287); PNPLA1 (NM_001374623); SDR9C7 (NM_148897); SLC27A4 (NM_005094), SULT2B1 (NM_177973); ST14 (NM_021978); TGM1 (NM_000359); ELOV4 (NM_022726), GJA1 (NM_000165), GJB3 (NM_024009), GJB4 (NM_153212), KDSR (NM_002035), KRT83 (NM_002282), PERP (NM_022121), TRPM4 (NM_017636), GJB2 (NM_004004), KRT1 (NM_006121), KRT10 (NM_000421), LOR (NM_000427), SPINK5 (NM_006846).

### In silico structural analysis

#### The sequence alignment

A blast homology search was performed using the program BLAST2SEQ available at the National Center for Biotechnology Information Website to compare amino acids sequences with wild-type sequences (https://blast.ncbi.nlm.nih.gov/Blast)). The evolutionary conservation of residues was performed using the Clustal Omega program (https://www.ebi.ac.uk/Tools/msa/clustalo/), by the alignment of the NIPA4 protein sequences of different species obtained from the Uniprot database (https://www.uniprot.org/uniprot/Q0D2K0) based on multiple sequence alignment (MSA).

#### Secondary structure prediction

The prediction of the secondary structure of NIPA4 protein was performed using Secondary Structure Prediction Server (APSSP) (http://crdd.osdd.net/raghava/apssp/). In addition, Protter server was used to predict the transmembrane helices (http://wlab.ethz.ch/protter/start/). The functional motif analysis (including the mutated amino acid residue) was performed using MOTIF search (http://www.genome.jp/tools/motif).

#### Structural protein modeling

To study the effect of a missense mutation on the structure of the human NIPA4 also known ichthyin protein encoded by the *NIPAL4* gene, we used a newly GPCR-I-Tasser described approach for the Human protein G modeling combining the homology modeling and the *ab-initio* prediction approach [33]. The amino acid sequence of the NIPA4 protein (NP_001092757) was obtained from the UniprotKB database (http://www.uniprot.org accession no: Q0D2K0) and no three-dimensional resolved structure was available in the Protein Data Bank. Previous works demonstrated that the NIPA4 protein, as ichthyin-like proteins share homology to both membrane Drug/Metabolite transporters (DMT superfamily) and G-protein-coupled receptors [34].

#### Molecular docking

Docking study was performed between the two NIPA4 proteins (wild-type and mutated p.Pro279Ala) and Mg^2+^ (ligand) using AutoDock 1.5.7rc1 [35]. The three-dimensional coordinates of each receptor were extracted from the resulting structures obtained following GPCR-I-Tasser modeling method as described above, the three-dimensional structures of Mg^2+^ were obtained from the PubChem Compound Organic Database in SDF format and then converted to PDB format using the PyMOL tool [51].

During the docking procedure, Mg^2+^ was used as the ligand, both proteins were held rigid. Grid maps representing the target proteins were constructed with different dimensions depending on the volume of the loop as well as the structural modification generated by the mutation at position 279 (S1 Table). The resulting ligand-protein complex interaction was visualized by Discovery Studio Visualizer 20.1.0 (BIOVIA, Discovery studio visualizer, San Diego, CA, USA, 2020).

#### Molecular dynamics simulation

A molecular dynamic (MD) study was carried out in order to examine the local three dimensional variation in the loop comprised between the two α- helices H5 and H6 including the p.Pro279Ala variant and to measure the conformational stability of the wild-protein NIPA4 with respect to the one mutated in position 279 (Pro279Ala).

The VMD software was used to generate the protein.psf files and the solvation box needed to initiate the MD. The molecules were solvated using the water box model that encompasses the entire proteins using VMD software [36–38].

Three steps were assessed for the wild-type protein and the p.Pro279Ala variant. The time step was set to 2 fs. The simulations were performed in the NpT conditions at a constant temperature of 310K at constant pressure 1, using the Langevin dynamics with a damping constant of 1 ps−1. Energy minimization was performed for 20,000 steps. After minimization step a second step water were equilibrated for 50 000 steps around proteins, which were restrained using harmonic forces with a spring constant of 2 kcal/(mol ˚A2). The last frames of restrained equilibration were used to start MD simulations for 140 ps were performed for both proteins (wild type/mutated) using Charm++ in NAMD [37, 39].

The root mean square deviation (RMSD) and root mean square fluctuation (RMSF) were calculated using the VMD tools [36, 38]. The plots were made using excel software.

## Results

### Case presentations and clinical history

**Fig 1:**
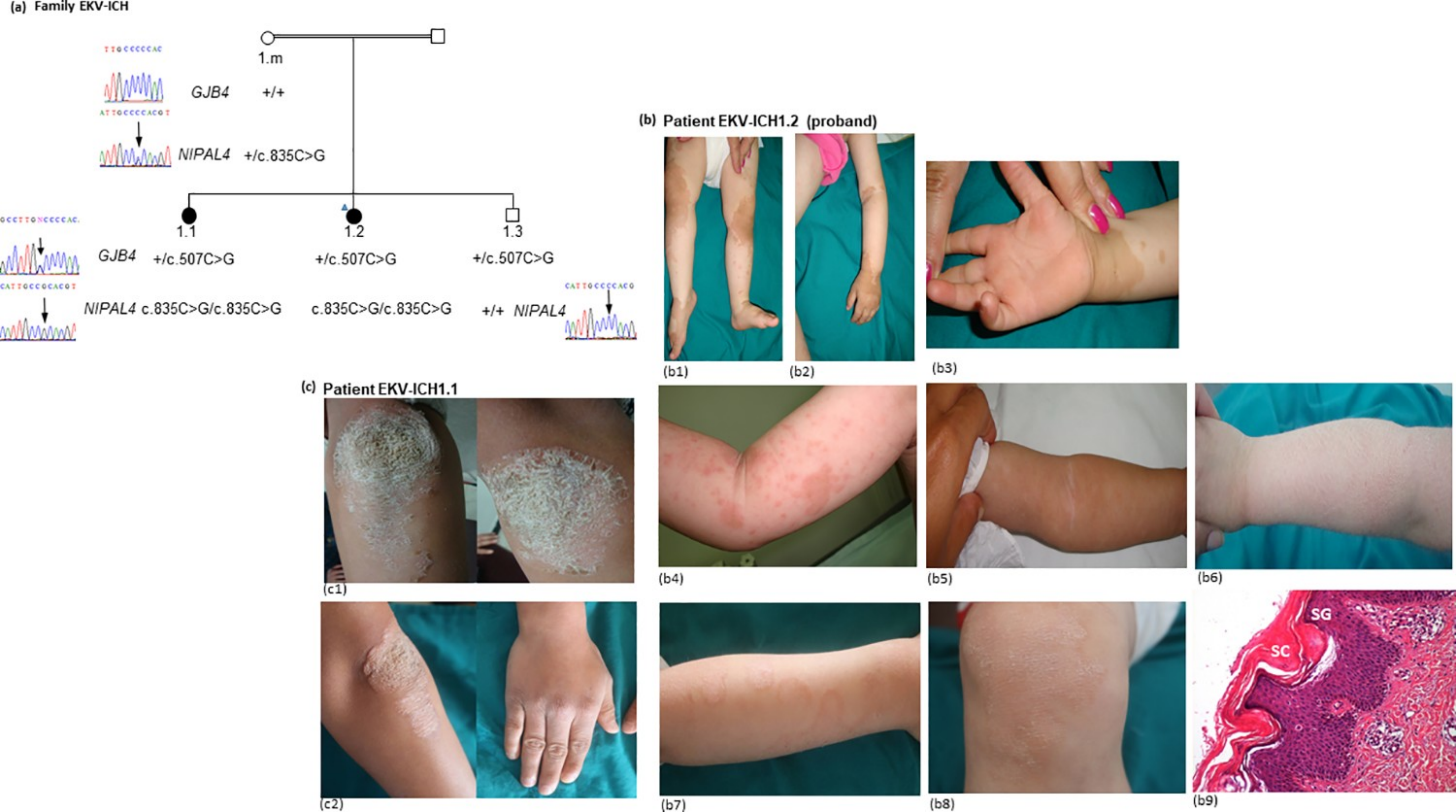
Pedigree, clinical and histological features of Erythrokeratodermia variabilis (EKV) in family EKV-ICH1. (a) Pedigree of the family segregating with autosomal recessive inheritance of EKV phenotype. The proband (EKV-ICH1.2) is indicated with a blue triangle. The sequence chromatograms illustrate the genotypes of tested family members for *GJB4* and *NIPAL4* genes. The likely polymorphism c.507C>G, (C169W) within the *GBJ4* gene (RefSeq NM_001005752) is identified at heterozygous state in both affected sisters (EKV-ICH1.1, 2) and the unaffected brother (EKV-ICH1.3). The c.835C>G (p.Pro279Ala) mutation within the *NIPAL4* gene (RefSeq NM_001099287) is identified at homozygous state in both affected sisters (EKV-ICH1.1, 2). Pedigree shows the cosegregation of the c.835C>G (p.Pro279Ala) mutation with EKV phenotype in a recessive mode. (b) The proband showed symmetric, well demarcated, brownish hyperkeratotic plaques localized on the knees, the feet (b1) and the dorsa of the hands (b2, b3) at the age of 18 months (first physical examination). Note the circinate borders of the erythematous plaques and the slight erythematous hyperkeratotic palmar keratoderma (b3). At the second visit, two weeks later, diffuse, migratory, figurative, erythematous spots and patches on the trunk and extremities (b4). Note the progression of the hyperkeratotic plaques and the variability of the distribution of hyperkeratotic plaques between the different clinical examination one month later (b5, b6). Widespread, large, adherent scale on extremities and trunk suggestive of ichtyosiform-lesions (b7). Two years later, fixed hyperkeratotic lesions on the forearms, knees, and dorsa of the hands (b7, b8). Histological findings from skin biopsy taken from the proband EKV-ICH1.2 showed hyperkeratosis associated with a compact, thickened stratum corneum (SC), hypogranulosis with a mild thinning stratum granulosum (SG) layer, papillomatosis, dilated follicles containing large keratin plugs, and a mild perivascular lymphocytic infiltrate (b9) (HE, x100). (c) The patient (EKV-ICH1.1) showed symmetric thick hyperkeratotic psoriasiform plaques over the knees at the age of 11 years. Two years later, symmetric hyperkeratotic squamous plaques with erythematous circinate borders on the elbows and erythematous and squamous plaques on the dorsa of the hands.

The proband, an 18-month-old girl (EKV-ICH1.2) was the second child of consanguineous parents originating from Northern Tunisia (Fig 1). She was referred to our Dermatology Department for the investigation of migratory erythematous and brownish plaques over the body. On inquiry, her mother (EKV-ICH1.m) revealed that these lesions started initially when her daughter was 5 months old. Over a period of 1 year, the lesions tented to change in shape and gradually migrate over time to various body areas frequently occurring on the lower extremities. A relatively sparing of the face was also noted. There was no history of collodion membrane at birth, alopecia, nail involvement and dental abnormalities in the family. The first physical examination of the proband (2016) revealed geographic, symmetric, well-demarcated, brownish, hyperkeratotic plaques localized mainly in the dorsa of the hands, wrists, elbows, knees, and the intertriginous areas (Fig 1b1 and 1b2). A mild erythematous scaly palmoplantar keratoderma was noted (Fig 1b3). The mucous membranes, scalp, hair, teeth and nails were normal, and no other physical abnormalities were noted. Two weeks later, the girl presented with migratory erythematous patches with circinate borders over the body (Fig 1b4) and extension of the hyperkeratotic pigmented plaques (Fig 1b5). A clinical diagnosis of EKV was made in view of the characteristic skin lesions and supported by the histological findings (Fig 1b9).

Based on this clinical presentation, and considering the age of the proband, topical application of 20% urea-containing keratolytics ointment combined with tretinoin cream 0.01% applied nightly were advised and followed by partial resolution of the hyperkeratotic plaques leaving a smooth surface and decreasing the itching sensation after 2 weeks.

Over a relatively short period, a diffuse xerosis was observed and the patient’s skin was covered by widespread, fine adherent scales, sparing the folds giving an ichtyosiform-like appearance (Fig 1b6).

During the last follow-up (until 2020), numerous new multiple, migratory, sharply demarcated, scaly, annular erythematous areas erupted on her four extremities in a diffuse pattern. Fixed hyperkeratotic lesions on the forearms, knees, and dorsa of the hands (Fig 1b7 and 1b8) were also noted. Erythema was induced by upset and warm conditions, and it can be accompanied by stinging or burning sensation.

#### Case EKV-ICH1.1

In 2016, during clinical assessment of the proband, the mother revealed that the elder sister (EKV-ICH1.1, Fig 1A) had a history of recurring and fluctuating migratory erythematous patches during childhood as her sister. There was spontaneous complete clearing of the eruption for several months followed by gradual progression in severity since 6 years of age. The mother also described that in contrast to her young, affected sister, the erythema progressively decreased in intensity, whereas diffusion of hyperkeratotic areas increased. The patient was seen by different physicians and was treated with various topical and fungal treatments that were not effective in controlling the disease.

At the time of this study, the elder sister (EKV-ICH1.1) was 11-years old when she was invited for further clinical evaluation. Examination disclosed massive, thickened and dark-coloured hyperkeratotic plaques over the knees and elbows for 2 months, leading to psoriasiform scales appearance (Fig 1c1). Her palms, soles, hair, nails, and teeth were not involved. The patient was instructed to apply keratolytics and topical corticosteroids once a day that was effective removing the scale within 10 days.

During the last follow-up (June 2020), the patient (EKV-ICH1.1) presented slowly progressive erythematous plaques symmetrically distributed on the elbows, dorsa of the hands and the knees (Fig 1c2). These hyperkeratotic plaques tended to be stabilized.

Audiometric studies and ophthalmological examination revealed no associated abnormalities in both patients. Normal clinical examinations were found for both the mother and the sibling (EKV-ICH1.3). The father did not show EKV or ichthyosis-like lesions throughout his lifetime according to his wife. Unfortunately, he refused to undergo clinical and genetic investigations. The family has limited access to medical care and become lost to follow-up.

### Absence of causative mutations in *GJB3* and *GJB4* known mutated EKV-related genes

The proband (EKV-ICH1.2) and her affected sister (EKV-ICH1.2) from family (EKV-ICH1.1) were initially screened for disease-causing mutations in the *GJB3* and *GJB4* genes, without finding causative mutations. This result further underlines the genetic heterogeneity of this disorder.

However, sequence analysis of the *GJB4* gene identified the c.507C>G, (C169W) at heterozygous state in both affected sisters and the unaffected brother. The mother did not present this nucleotide change, suggesting that it must be paternally inherited (Fig 1A). The pathogenic role of (C169W) mutation was previously described in various population associate with hearing impairment phenotype [40]. However, several investigations have concluded that C169W most likely represents a polymorphism [12, 41, 42]. According to the gnomAD database, the C169W variant numbered rs755931 (GRCh37) in dbSNP, is very common in African (frequency 26.4%) and East Asian (frequency 13%) populations. Additionally, we checked for the occurrence of the C169W in 50 in-house control exomes. The C169W was found in heterozygote state (CG) (2/50 (4%)) and in homozygote state (GG) (3/50 (6%)), this proved that this mutation represents a common neutral polymorphism in Tunisian population. Thus, C169W is less likely to have a contributory effect on EKV phenotype in this case.

### Novel missense mutation in *NIPAL4* gene

Sample from the proband (EKV-ICH1.2) was subjected to WES, in order to identify the genetic defect underlying the EKV phenotype. Given the known consanguinity in this family and the likely autosomal recessive mode of inheritance according to the pedigree, we expected a homozygous as well as compound heterozygous disease-causing variants. Meanwhile, the occurrence of ichthyosiform-like lesions in the proband leads us to investigate known causative genes for distinct forms of inherited dermatoses with erythematous and hyperkeratotic components including non-syndomic ARCI and erythrokeratodermias, more specifically EKVP. In view of the variability and the overlap clinical manifestations of erythrokeratodermias disorders, we also analyzed the genes that have been previously implicated in the clinical differential diagnosis of EKVP. The list of the prioritized candidate genes including a total of 28 genes was screened for putative pathogenic variants: the 14 genes known as the most common mutated genes (*ABCA12*, *ALOX12B*, *ALOXE3*, *CASP14*, *CERS3*, *CYP4F22*, *LIPN*, *NIPAL4*, *PNPLA1*, *SDR9C7*, *SLC27A4*, *SULT2B1*, *ST14*, *TGM1*) for about 85% of non-syndromic ARCI cases, the 8 relevant genes (*ELOV4*, *GJA1*, *GJB3*, *GJB4*, *KDSR*, *KRT83*, *PERP*, *TRPM4*) involved in the EKVP phenotypes and the 6 already known genes (*ABHD5*, *GJB2*, *KRT1/KRT10*, *LOR*, *SPINK5*) to be associated with the clinical differential diagnosis of EKVP (Table 1). The summary of the bioinformatics workflow of this study is presented in Table 2. The filtering/prioritization pipeline resulted in 30,428 rare potential functionally relevant variants, among them 42 were present in analysed candidate genes. Out of those, only one homozygous nonsynonymous variant (g.157472394C>G; c.835C>G; p.(Pro279Ala)) in exon 6 of *Nipa-Like Domain-Containing 4* (*NIPAL4/Ichthyin*, RefSeq NM_001099287, 5q33.3) is predicted to be deleterious and damaging according to SIFT, PolyPhen2, MutationTaster, PROVEAN, MetaSVM, MetaLR, MutationAssessor, fathmm-MKL and CADD (25.4) prediction tools. Direct sequencing confirmed the presence of this same genetic variant in homozygous state in the older affected sister (EKV-ICH1.1), in heterozygous state in unaffected mother (EKV-ICH1.m) and its absence in the unaffected sibling (EKV-ICH1.3). Therefore, (*NIPAL4*, c.835C>G p.(Pro279Ala) could possibly represent a potential disease-causing variant of EKV phenotype most likely inherited in an autosomal recessive pattern. In addition, the *NIPAL4* gene has never been involved in EKV phenotype according to the currently literature.

**Table 2:** Number of variants at the different stages of filtration in the exome sequencing data of the proband (EKV-ICH1.2).

Total number of variants	**105,744**
Number of functionally relevant variants (Exonic, splicing, 3’UTR, 5’UTR, upstream, downstream)	**30,428**
Number of variants in prioritized genes	**42**
Number of predicted deleterious variants (SIFT, POLYPHEN2, Mutation Taster, Mutation Assessor, FATHMM, PROVEAN, MetaSVM, MetaLR)	**36**
Number of deleterious variants in ClinVAR	**3**
Number of deleterious variants in prioritized genes	**1 *NIPAL4*** (RefSeq NM_001099287) (ch 5: g.157472394C>G ; c.835C>G; p.(Pro279Ala)) (exon 6)*

* The novel identified mutation (g.157472394C>G; c.835C>G; p.(Pro279Ala) in exon 6 of Nipa-Like Domain-Containing 4 (NIPAL4/Ichthyin, RefSeq NM_001099287) has been submitted in ClinVar database under the accession number (SCV001733618.1) and reported in https://www.ncbi.nlm.nih.gov/medgen/C4551486/.

Compound heterozygosity in all the prioritized genes was also checked without finding convincing results (S2 Table). On theses bases, the (c.835C>G p.Pro279Ala) variant has been classified as “likely pathogenic” supporting by the ACMG pathogenicity criteria PM2, PM5, PP2 and PP3. Thus, we considered that the mutation of *NIPAL4* was probably the relevant genetic cause of the disease phenotype in the EKV-ICH1 family.

### Computational analysis of *NIPAL4* variant

#### Structural effect of proline 279 mutation on NIPA4

**Fig 2:**
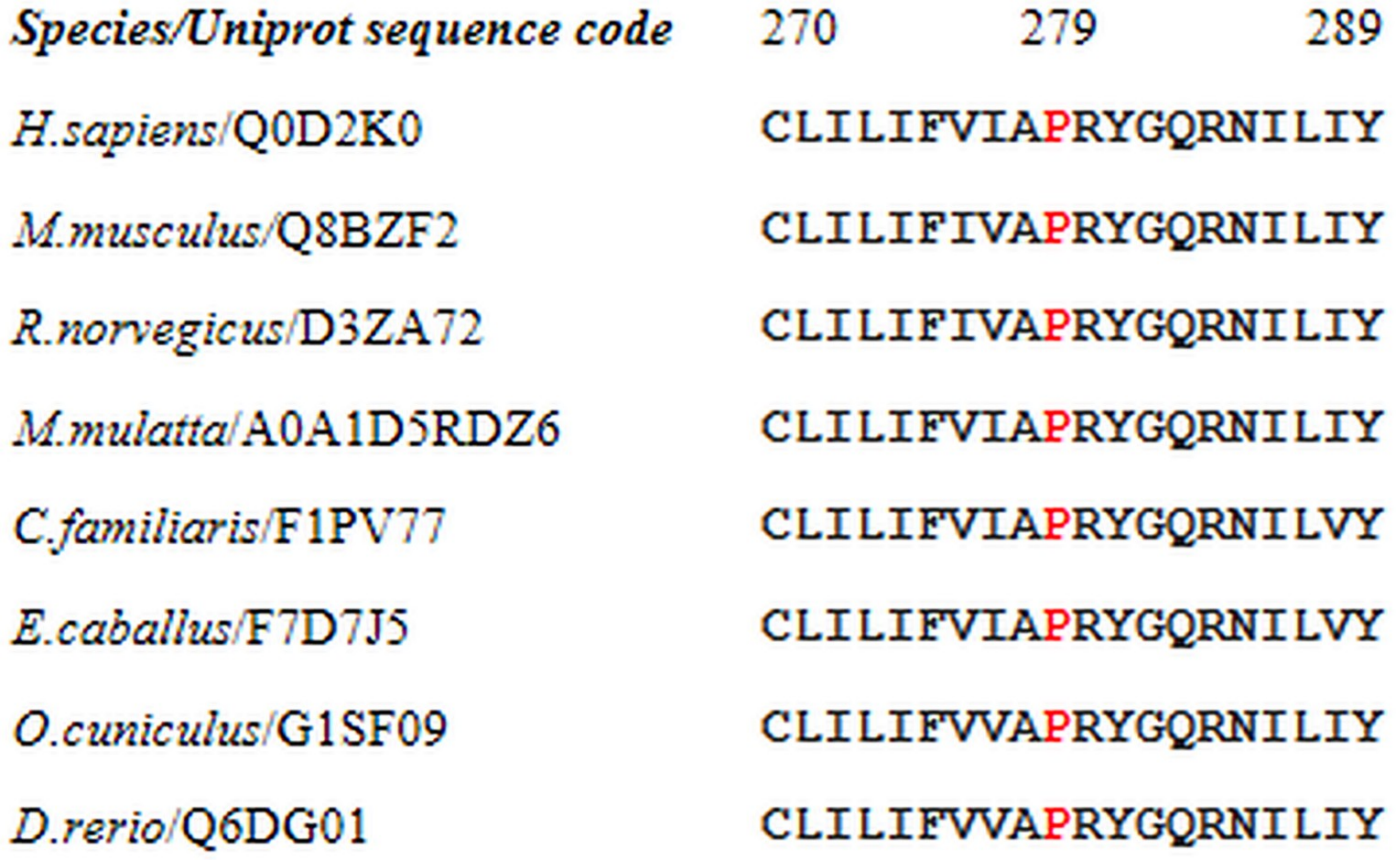
Multiple alignment of the NIPA4 protein sequences in different species showing the conserved nature of the proline residue at position 279 throughout species.

Multiple sequence alignments of NIPA4 protein were performed by comparing several sequences of the NIPA4 protein obtained from the different target species available in the Uniprot database via Clustal Omega Tools (https://www.ebi.ac.uk/Tools/msa/clustalo/). Our results showed that the proline at position 279 is an evolutionarily residue across different species, providing evidence of the crucial function of this amino acid residue (Fig 2).

The conserved proline residues (P) at position 279 are in red color. The letters represent the single-letter codes for amino acids. The c.835C>G p.(Pro279Ala) (NP_001092757) variant is included in the A-P-R-Y-G-Q-R-N motif and occurred in the predicted region connecting the helix 5 to transmembrane helix 6 according to Protter. Three motifs were introduced into the MOTIF2 routine, the first corresponds to the native sequence of NIPA4 (A-P-R-Y-G-Q-R-N), the second motif containing a random amino acid (A-x-R-Y-G-Q-R-N), x corresponds to the position 279 containing the mutated variant, finally the third pattern corresponds to the new pattern including the mutated Ala residue (A-**A**-R-Y-G-Q-R-N). The three patterns were tested in different protein databases (GenBank, UniProt, RefSeq and PDBSTR).

**Fig 3:**
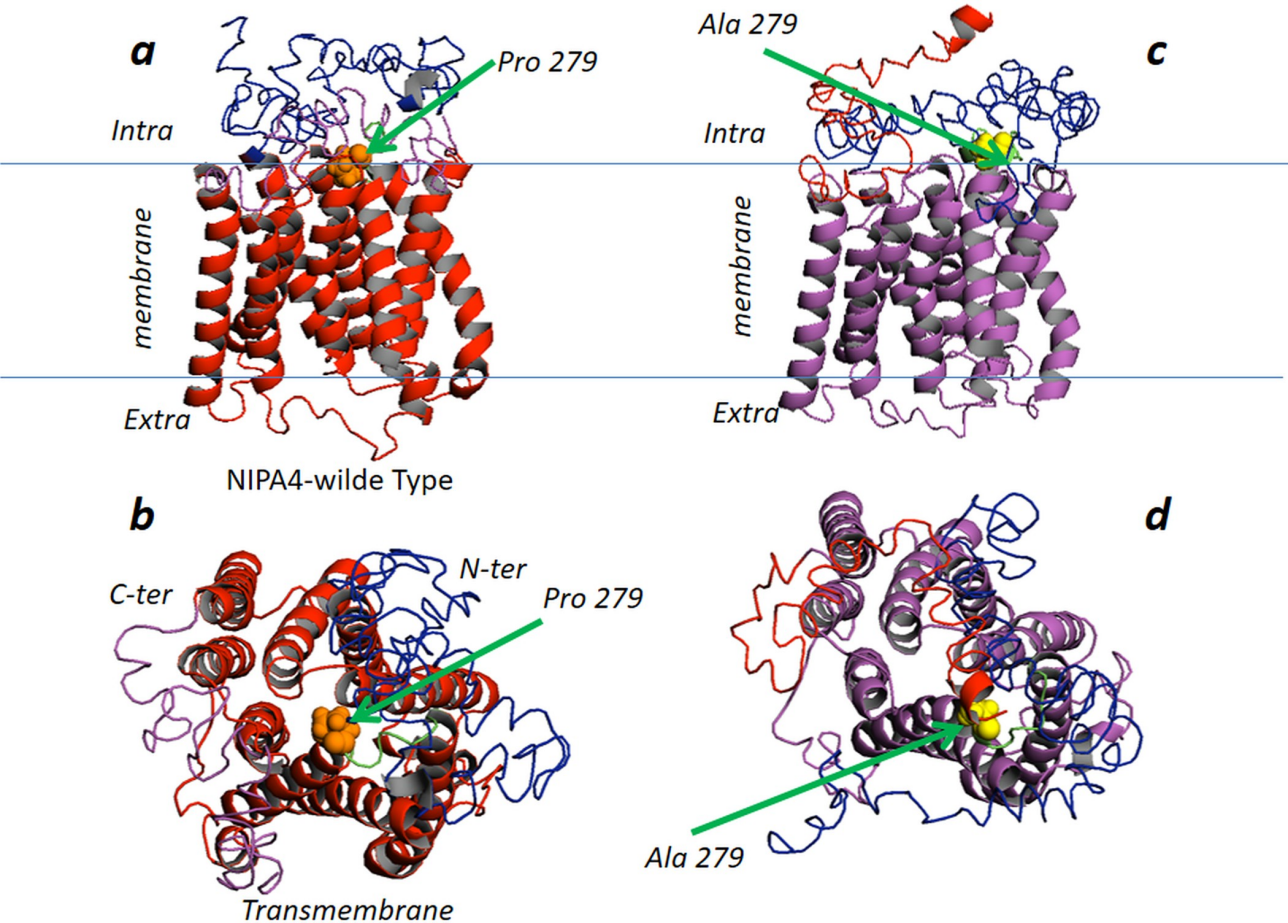
Structural representation of the wild-type and mutated p.(Pro279Ala) NIPA4 protein. Left: top and bottom; Two perpendicular views (a and b) are shown. Top (a): Side view in ribbon cartoon of the wild-type. The helical transmembrane region colored in red, the N-terminal region colored in magenta, the C-terminal region colored in blue, proline 279 in CPK and colored in orange. The central transmembrane core of NIPA4 protein (amino acid: 116–411) is formed by nine transmembrane α-helices noted H1 to H9 (H1 (116–140); H2 (162–186); H3 (190–211); H4 (217–237); H5 (251–277); H6 (285–313); H7 (322–349); H8 (353–374); H9 (380–411) and a random coiled N- (1–115) and C-terminal (412–466) regions. This allows flexibility to the N-terminus and C-terminus regions independently of the transmembrane region. The model of NIPA4 protein was built based on herpesvirus fusion regulator complex gH-gL (PDB: 3m1c) and the structure of the membrane transporter YddG (PDB: 5i20) belonging to the large superfamily of the ubiquitous drug/metabolite transporter, export drugs and metabolites (DMT) [43]. The NIPA4 model presents a similar fold and topology of the template: the nine α-helices are organized like the predicted transmembrane helices, with a large substrate-binding cavity at the center of the receptor. The bottom view (b) highlights the intracellular gate and the cavity that accommodates the Mg^2+^ ion channel. Right: top and bottom (c and d); Two perpendicular views are shown. Top (c): Side view in ribbon cartoon of the p.Pro279Ala mutation. The helical transmembrane region colored in magenta, the N-terminal region colored in blue, the C-terminal region colored in red, Ala 279 in CPK and colored in yellow. The bottom view (d) highlights the intracellular gate and the cavity that accommodates the Mg^2+^ ion channel.

The prediction of the 3D structure of NIPA4 was performed by GPCR-I-Tasser server using the best alignment template obtained from Protein Data Bank database. Five models were generated with C-scores ranging from -4.51 to -2.89 for the NIPA4 wild -type and −4.67 to -2.33 for the p.Pro279Ala mutation. The one with the highest C-score represents the best model. The highest values for predicted models of the wild-type and the missense mutation p.Pro279Ala were −3.72, and −3.79 respectively. Those models were selected for this study. Predicted models for NIPA4 were described in Fig 3A and 3B.

According to the structural prediction by GPCR-I-Tasser, the protein wild-type is located in the transmembrane region with a cytoplasmic N-terminal extremity and extracellular C-terminal one. Nine transmembrane helices were displayed by the GPCR-I-Tasser program as predicted by Phobus, Protter and Uniprot servers. The overall model structure showed the potential transport channel delimited by the transmembrane helices (Fig 3B).

The 3D model structure of the p.Pro279Ala mutation of NIPA4 protein is well superimposed on the wild-type protein except the turn linking H5 to H6 helices (Fig 3C and 3D). In the wild-type protein, Pro279 is located at the end of the predicted transmembrane H5 of the hypothetical transport channel cavity that may play a key role in the function of the channel. This suggestion is reinforced by the model designed and by the conservation of the 279 amino acid in most of the protein sequences of the NIPA4. More precisely, Pro279 forms the first residue of the turn connecting the transmembrane helices H5 and H6. This turn is also highly conserved between species suggesting a key role in the function of the NIPA4 protein.

**Fig 4:**
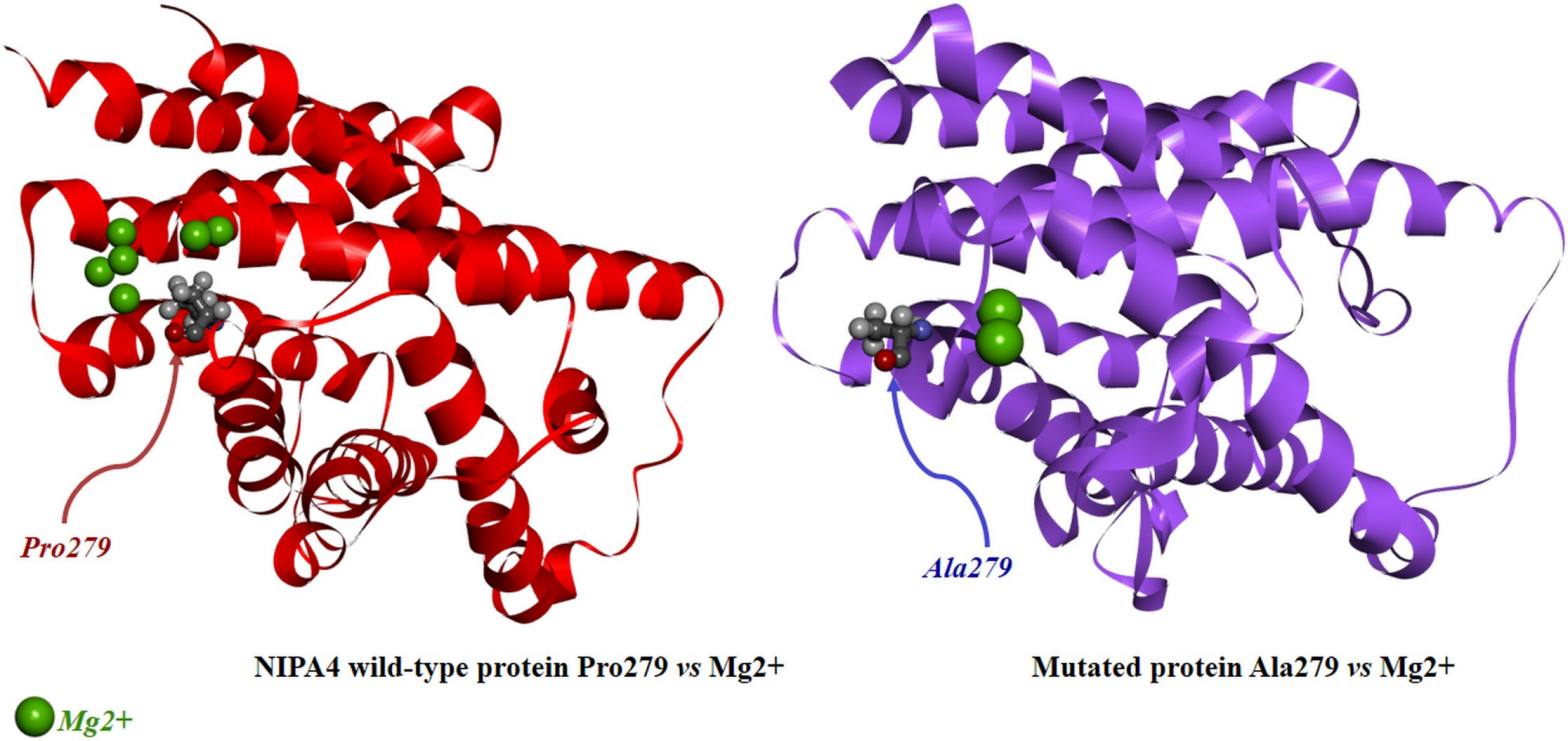
Docking analysis of the wild-type and the mutated p.(Pro279Ala) NIPA4 proteins against Mg^2+^.

In the p.Pro279Ala mutant protein model, H5 is elongated by three residues including alanine 279 (Fig 3C). The p.Pro279Ala mutation seems to shift and reduce the turn connecting the predicted transmembrane helices H5 and H6. Consequently, the end of the channel becomes accessible, which in turn could disrupt the proper functioning of the channel and alter the flow of Mg^2+^ in the absence of a regulator. In order to further demonstrate the effect of the p.Pro279Ala mutation on the Mg^2+^ flow, docking study was carried out via AutoDock tool considering the wild-type and the mutated NIPA4 proteins against Mg^2+^. As shown in the Fig 4, the presence of proline at position 279 promotes the formation of a loop for which the conformational status has a higher affinity for the Mg^2+^ ions compared to the loop including the alanine at position 279 in the NIPA4 mutated protein. This conformation change induces the reduction of the six binding sites of Mg^2+^ ions observed in the wild-type protein to only two sites in the mutated protein, leading to the alteration of the Mg^2+^ flow trough the NIPA4 potential channel due to the p.Pro279Ala mutation (Fig 4).

#### Molecular dynamics simulations of wild-type and mutated NIPAL4

**Fig 5:**
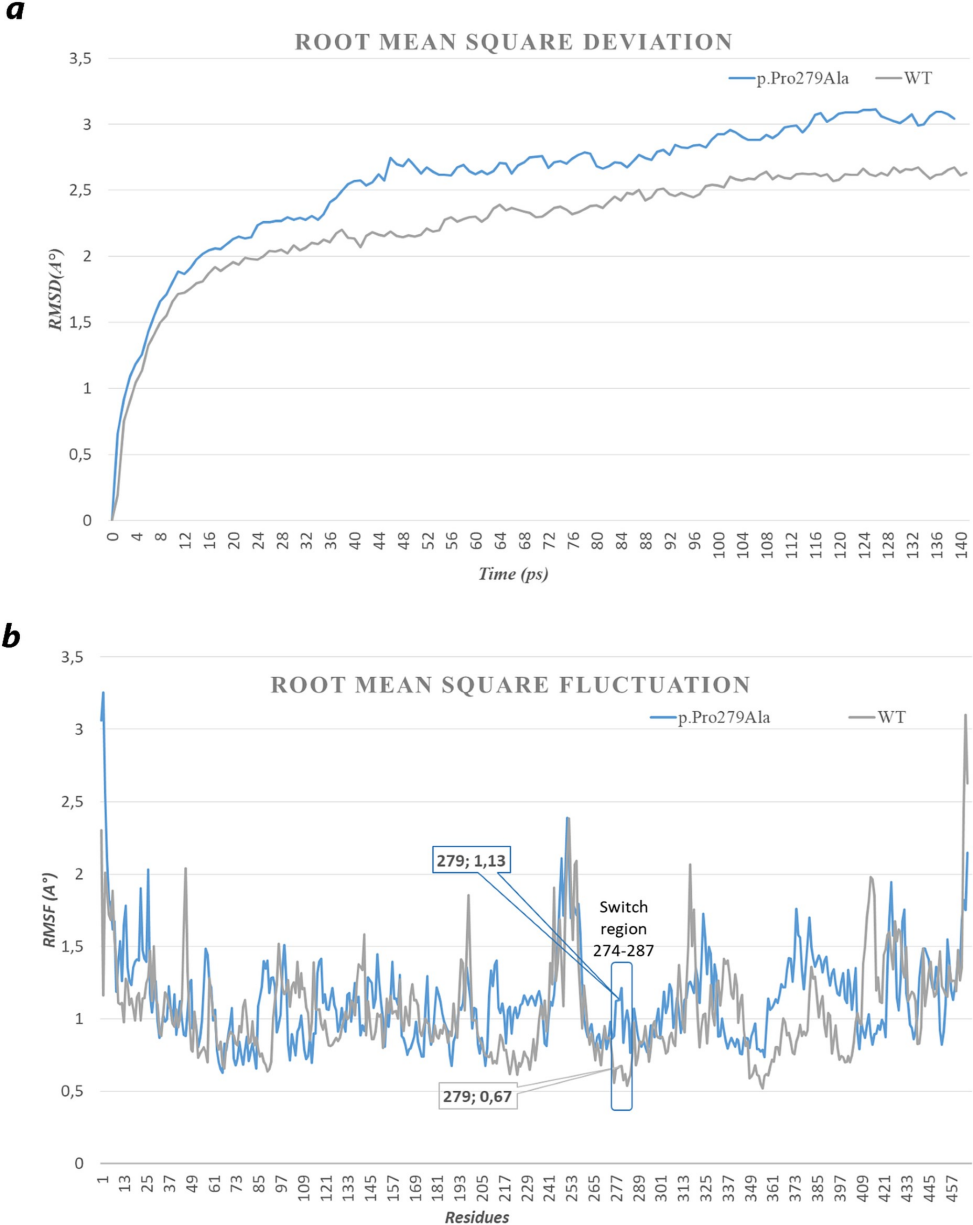
Analysis of simulation trajectory of the wild-type and mutated (p. Pro279Ala) NIPA4 protein for 140 ps. (a) Time evolution of backbone RMSD as a function of time, (b) RMSF of the Cα atoms as a function of residue. A lesser value was found in RMSD for the wild-type (blue) than that the mutant protein (grey indicating a more stability for the wild-type than the altered protein. These results indicate that the flexibility of NIPA4 was improved due to the mutation on Pro279Ala. The superimposition of the RMSF of all amino acids of the wild-type with the p.Pro279Ala mutation indicates a strong modification in the region containing the mutation. This region was surrounded by a rectangle in (b). The fluctuation of the loop (274–287) clearly demonstrates the mobility of the mutated region compared to the wild-type, probably leading to the dysfunction of the Mg^2+^ ion channel.

We performed molecular dynamics (MD) simulations of the wild-type and mutated NIPA4 protein in order to examine the structural consequence and the stability aspects of the p.Pro279Ala mutation. The potential of each trajectory produced was thoroughly analyzed after MD simulations. Once the 70,000 calculation cycles were completed, 140 frames corresponding to 140 ps (time of stimulation) were obtained for the native protein as well as for the mutated protein. The RMSD plot is displayed in Fig 5A. With the aim of determining whether the mutation affects the dynamic behavior of residues, the RMSF values of wild-type and mutant structures were compiled. The superimposed RMSF plots of the wild-type NIPA4 and mutated protein show a fluctuation pattern that does not exceed 3.5 Å for the majority of the residues (Fig 5B). The difference was visualized at residues 277–280 where the fluctuation is no more than 0.7 A° for the wild-type protein while for the mutated protein values above 1.2 A° were obtained. Otherwise, Pro279 in the wild-type protein shows an RMSF of 0.672A° compared to Ala279 in the mutated protein which shows an RMSF of 1.260A°.

The RMSD variation of the wild-type protein predominates in the range of 0.196 to 2.672Å. However, for the mutated protein, it ranges from 0.660A° to 3.112 Å. The wild-type protein showed deviations during the initial simulations up to about 100 ps but over time it was stable around 2.5 Å while the mutated protein shows a strong variability and the RMSD values remain increased throughout the simulation.

## Discussion

To the best of our knowledge, we describe here the first clinical and molecular report of a Tunisian family with 2 sisters displaying clinical features of EKV. Migratory shaped erythematous areas are the initial presenting sign followed by relatively stable hyperkeratotic plaques are the two predominates characteristics in both patients. However, remarkable variability of morphological and dominating features of the disease were observed between the two affected sisters, but also during the disease course of each individual patient.

In the older sister, the erythematous lesions become fixed, thickened symmetric hyperkeratotic plaques largely confined to areas exposed to mechanical stress. While, in the younger patient, the erythematous lesions remain migratory accompanied by relatively stable hyperkeratotic plaques on the extremities.

The clinical pattern of the younger patient (proband) showed the coexistence of migratory erythematous component with scaly, annular patches and the manifestation of a diffuse xerosis clinically resembling ichthyosiform-like lesions, distinct from those in classical EKV. Although, annular erythematous lesions with scaling have been described only rarely in patients with typical features of EKV [6], the ichthyosis appearance raised the question of whether the EKV phenotype exhibited by the younger patient is distinct from ichthyosiform genodermatoses that may involve erythematous and hyperkeratotic components [18, 44]. The annular epidermolytic ichthyosis (MIM 607602) variant of bullous congenital ichthyosiform erythroderma was excluded based on the absence of epidermolytic hyperkeratosis in histology [45]. Keratitis-ichthyosis-deafness syndrome (MIM 148210) was also ruled out because the absence of characteristic clinical criteria (i.e, alopecia, sensorineural hearing loss, photophobia, corneal vascularization), as well as the absence of histological findings of cobblestone-like hyperkeratosis. The neutral lipid storage disease with the clinical appearance of ichthyosis (Chanarin-Dorfman syndrome (MIM 275630)) was excluded based on the absence of lipid droplets in basal and granular keratinocytes [20]. Netherton syndrome (MIM 256500) is considered the most frequent ichthyosis syndrome, it is typically characterized by migratory, serpiginous red plaques with double-edged scaly borders. However patients usually have other manifestations including hair shaft abnormalities that are not observed in the studied case [46]. Loricrin keratoderma (MIM 604117) designed as ichthyotic variant form of Vohwinkel’s syndrome without hearing loss is clinically characterized by erythematous hyperkeratotic plaques similar to those seen in PSEK but it differs by the presence of mutilating palmoplantar keratoderma [32]. The possible diagnosis of loricrin keratoderma is unlike due to the lack of palmoplantar keratoderma with pseudoainhum in the proband. Hence, the clinical presentation of family EKV-ICH1 may probably be a phenotypic variant of EKV with ichthyosiform appearance in one affected member.

Mutational screening initially performed of the *GJB3* and *GJB4* genes underlying EKV failed to disclose pathogenic mutations in the studied family, confirming further genetic heterogeneity of the condition. This result also suggests that mutations in another gene could give rise to a similar phenotype of EKV involving ichthyosiform erythrokeratodermia. We subsequently decided to perform WES for the proband. Our analysis focused on variants located in a list of the most common mutated genes associated with ARCI, EKVP and the differential diagnosis of EKVP taking into consideration the proband’s phenotype (Table 1).

The ARCI is a rare, clinically, and etiologically heterogeneous group of cornification. It defines three clinical subtypes which include the spectrum of Lamellar Ichthyosis (LI), Congenital Ichthyosis Erythroderma (CIE°) and Harlequin Ichthyosis (HI). It is mainly characterized by localized or generalized hyperkeratosis and scaling, often accompanied by erythema, fissures and erosions [47, 48]. In most cases of ARCI, infants are born with a collodion membrane. Over the last decade at least ten different genes have been implicated in the etiology of ARCI [29, 30]. All of these genes encode proteins that are involved in the formation of the cornified lipid envelope in the stratum corneum and ceramide formation and processing in the epidermis [49]. With the exception of the severe HI, which is mainly due to *ABCA12* mutations, LI and CIE phenotypes may occur as a consequence of pathogenic variants in most of the known ARCI genes, thereby making difficult clinical and molecular classification of the different subtypes of ARCI [50].

In this study, WES analysis revealed a novel likely pathogenic homozygous nonsynonymous variant (c.835C>G, p.Pro279Ala) within *NIPAL4* gene in family EKV-ICH1. *Nipa‐Like Domain‐Containing 4* (*NIPAL4* also known as ICHTHYIN) is the second most commonly mutated gene in ARCI [47, 51]. The NIPA4 works as Mg^2+^ transporter, which appears to be involved in epidermal lipid processing and the lamellar body formation and traffic [47]. However, the role of NIPA4 in skin barrier formation and the mechanism of NIPA4-mediated ARCI pathogenesis remain unclear [52]. Mutations in *NIPAL4* gene are associated with the phenotypes of both LI and CIE. The scales are large, adherent, dark and pigmented, with an absence of skin erythema in LI. In contrast, in CIE, the scales are fine and white on an erythematous background, although they are larger and grayish on the limbs [48]. Despite this clinical distinction, clinical overlap can occur between LI and CIE, and patients frequently have manifestations of both conditions in addition to other features, such as nail clubbing, alopecia and ectropion. LI is considered as more severe than CIE. The presence of collodion membrane at birth is variable and can be absent in up to 60% of patients with ARCI linked to *NIPAL4* mutations [34].

In this study, the clinical features of the proband present partial similarity to those of the phenotype associated with *NIPAL4* mutations including fine, brown scaling with variable erythroderma and mild palmoplantar keratoderma. However, they differ by the absence hypohidrosis or ectropion and the absence of generalized skin scaling that are major manifestations in patients with ARCI harboring *NIPAL4* mutations. Neither of the two affected sisters in our study was a collodion baby at birth (Table 3).

**Table 3:** Specific clinical symptoms of EKV and ARCI reported in the literature and clinical features of patients EKV-ICH1.1 and 2.

**Common clinical symptoms to EKV**	**Patient EKV-ICH 1.1**	**Patient EKV-ICH1.2**
Generalized hyperkeratosis	-	-
Localized hyperkeratosis	+	+
Fixed keratosis	++	+
Palmoplantar Keratoderma	+	+
Variable, transient erythema	-	++
Xerosis with fine scale	-	++
Hyperpigmentation	-	++
Hypertrichosis	-	-
Trunk lesions	-	++
Flexural predominant	-	++
Facial lesions	-	-
Others		
Circinate erythema	+	+
Gyrate erythema	+	++
**Common clinical symptoms to ARCI**	**Patient EKV-ICH 1.1**	**Patient EKV-ICH 1.2**
Collodion baby	-	-
Scaling type/color	+ (thick gray scales on the elbows and the dorsa of the hands)	+++ (large brown scales on extremities, forearms, knees and trunk)
Erythroderma	-	-
Keratoderma	+	+
Diffuse yellowish keratoderma	-	-
Ectropion	-	-
Eclabium	-	-
Alopecia	-	-
Heat intolerance	-	-
Hyperlinearity	+	+
Clubbing of nails	-	-
Moucosae	-	-
Photophobia	-	-
Lacrimination	-	-

+ patient positive for characteristic, ++ pronounced effect, +++ intense effect, − characteristic not observed.

So far, 34 disease‐causing mutations have been reported in *NIPAL4* including the highly recurrent mutation c.527C>A, p.(Ala176Asp) which is a possible hot spot [29, 34, 53–57]. Recently, a large international cohort of 101 families affected with ARCI carrying mutations in *NIPAL4* was reported, among them 25 families were from Morocco, Algeria and Tunisia and 7 families were from Turkey and Syria [29]. This report in accordance with previous studies suggest that ARCI phenotype associated with *NIPAL4* is relatively frequent in the Mediterranean basin particularly in North Africa probably in relation with the high rate of consanguinity in those populations [58]. In the same large international cohort, a French patient genotyped p.(Ala176Asp)/p.(Pro279Leu) involving the identical mutated codon Pro279 as described here, exhibited moderate LI phenotype with erythema. There are no available detailed clinical and histological findings of this patient to establish the phenotypic spectrum associated with the Pro279 mutation.

To evaluate the effect of the p.(Pro279Ala) mutation, we performed a comprehensive analysis of the structural change of the NIPA4 protein. Initially, the mutation affected a highly conserved residue. Next, the predicted 3D structure of NIPA4 protein revealed that the p.Pro279Ala mutation is located at the end of the transmembrane α-helix H5 of the hypothetical transport channel cavity that could affect its Mg^2+^-transporter activity in skin cellular compartments (Fig 3A). *In silico* analysis predicted that the Pro279 would probably act as a regulator of the inflow and outflow of Mg^2+^ cations due to its cis/trans isomerization effect. Therefore, the p.(Pro279Ala) mutation may lead to an alteration of the conformational change on the ion channel. Molecular dynamics simulation study has further supported our hypothesis and provided a detailed idea about the structural aspects of the p.(Pro279Ala) and its effects on NIPA4. It is shown that the occurrence of the mutation leads to a more flexible conformation compared to the wild-type structure.

Therefore, all above-mentioned facts support the possibility that a defect in NIPA4 might disturb its transporter function, which in turn might lead to an alteration of pathways requiring NIPA4 as an Mg^2+^ recruitment. Previous studies have demonstrated the involvement of NIPA4 in transportation of epidermal lipids processing that is essential for maintaining the permeability and barrier function [59]. The histological findings of hyperkeratosis and reduced intercellular gaps in the cornified layers of the epidermal skin of the proband EKV-ICH1.2 demonstrated disturbance of keratinocytes differentiation as well as impairment of the intercellular lipid layers (Fig 1b9). The defective formation of the lipid layers is thought to result in serious loss of the epidermal barrier function and to abnormal hyperkeratosis. Interestingly, histological analyses in the epidermis of Nipal4-knockout mice model showed several morphological abnormalities including hyperkeratosis, impairment of lipid multilayer structure formation, immature keratohyalin granules as well as decreasing level of the skin barrier lipid acylceramide that further support the histological observations in the proband [52]. Hence, the defective NIPA4 may result in the formation of an abnormal stratum corneum, the outermost epidermal layer with defective intercellular lipid layers, thus leading to compromised permeability barrier functions and ARCI-like manifestations observed in the proband [60]. Furthermore, it has demonstrated that Mg^2+^ concentration in differentiated Nipal4- knockout mice keratinocytes was reduced compared than that to the wild-type keratinocytes, causing aberration in the proper chromatin remodeling process, which in turn leads to failure of differentiation-dependent gene induction in keratinocytes [52]. It is highly likely that the reduction of the Mg^2+^ binding sites in the mutated NIPA4 protein caused by the p.(Pro279Ala) mutation and supported by the docking study, might explain the clinical presentation of ichthyosis observed in the proband.

## Conclusion

Overall, this result expands the current knowledge of the clinical phenotype associated with *NIPAL4* mutation.

While additional patients are needed to fully delineate the phenotype of the disease and its underlying mechanism, the family presented in this report supports the existence of a recessive form of EKV-like ARCI related to an ichthyosis gene. This study provides additional insights into the value of precise clinical evaluation and a patient follow-up in combination with WES approach to identify the causative gene and variant associated with a specific phenotype of very rare inherited skin disorder characterizing by genetic heterogeneity, overlapping phenotypes, and with causal genes involved in common disease-related pathways. Nevertheless, future consideration must also be given to other types of variants, such as copy number variations (CNVs), which could influence gene expression and thereby phenotypic variation, thus providing new and clinically relevant insight into the EKV disease. Further experiments are also needed to determine the function role of NIPA4 in keratinocytes differentiation and the relationship in the pathogenesis of ichthyosiform dermatosis.

## Supporting information

S1 Table Different dimensions of the Grid box according to the volume of the loop containing the mutation in position 279. (DOCX)

S2 Table Additional WES data of patient EKV-ICH1.2. (XLSX)
