# Supplementary material for: Expanding the clinical phenotype associated with NIPAL4 mutation: Study of a Tunisian consanguineous family with erythrokeratodermia variabilis—Like Autosomal Recessive Congenital Ichthyosis
Source: PLoS One. 2021 Oct 20;16(10):e0258777. doi: 10.1371/journal.pone.0258777 (PMC8528321; doi:10.1371/journal.pone.0258777)
Supplement: S1 Table — (DOCX) [file pone.0258777.s001.docx]

**S1 Table. Different dimensions of the Grid box according to the volume of the loop containing the mutation in position 279.**

| **Protein** | ***Grid maps dimensions*** | | | | | |  |
| --- | --- | --- | --- | --- | --- | --- | --- |
|  | ***X_-Dimension_*** | ***Y_-Dimension_*** | ***Z-_Dimension_*** | ***X_-center_*** | ***Y_-center_*** | ***Z_-center_*** | ***Spacing*** |
| **wild-type protein** | 30 | 28 | 32 | 68 | 63 | 70 | 1.000 A° |
| **p.Pro279Ala** | 44 | 24 | 40 | 75 | 70 | 68 |  |
